# State of the Art Bowel Management for Pediatric Colorectal Problems: Functional Constipation

**DOI:** 10.3390/children10061078

**Published:** 2023-06-19

**Authors:** Elizaveta Bokova, Wendy Jo Svetanoff, John M. Rosen, Marc A. Levitt, Rebecca M. Rentea

**Affiliations:** 1 Comprehensive Colorectal Center, Department of Surgery, Children’s Mercy Hospital, Kansas City, MO 64108, USA; 2 Division of Pediatric Gastroenterology, Hepatology, and Nutrition, Children’s Mercy Kansas City, Kansas City, MO 64108, USA; 3 Department of Pediatrics, University of Missouri-Kansas City, Kansas City, MO 64108, USA; 4 Division of Colorectal and Pelvic Reconstruction, Children’s National Medical Center, Washington, DC 20001, USA; 5 Department of Surgery, University of Missouri-Kansas City, Kansas City, MO 64108, USA

**Keywords:** bowel management, functional constipation, botox, botulinum toxin, fecal incontinence, enema, laxatives, constipation, colorectal surgery, resection

## Abstract

Background: Functional constipation (FC) affects up to 32% of the pediatric population, and some of these patients are referred to pediatric surgery units to manage their constipation and/or fecal incontinence. The aim of the current paper is to report the recent updates on the evaluation and management of children with FC as a part of a manuscript series on bowel management in patients with anorectal malformations, Hirschsprung disease, spinal anomalies, and FC. Methods: A literature search was performed using Medline/PubMed, Google Scholar, Cochrane, and EMBASE databases and focusing on the manuscripts published within the last 5–10 years. Results: The first step of management of children with FC is to exclude Hirschsprung disease with a contrast study, examination under anesthesia, anorectal manometry (AMAN). If AMAN shows absent rectoanal inhibitory reflex, a rectal biopsy is performed. Internal sphincter achalasia or high resting pressures indicate botulinum toxin injection. Medical management options include laxatives, rectal enemas, transanal irrigations, and antegrade flushes. Those who fail conservative treatment require further assessment of colonic motility and can be candidates for colonic resection. The type of resection (subtotal colonic resection vs. Deloyer’s procedure) can be guided with a balloon expulsion test. Conclusion: Most of the patients with FC referred for surgical evaluation can be managed conservatively. Further studies are required to determine an optimal strategy of surgical resection in children unresponsive to medical treatment.

## 1. Introduction 

Functional constipation (FC) affects up to 32% of children, with a higher frequency in toddlers [1,2,3,4,5]. The condition is a high burden on the U.S. healthcare system contributing to 10% of emergency department visits for abdominal pain and 10–25% of gastroenterology consults [6,7,8] with 2.5 million physician visits in the United States per year [9,10]. Half of the children with functional defecation disorders managed by a gastroenterologist have persistent symptoms 5 years after referral, and 10% are still constipated at a 10-year follow-up [11], with one-third of children remaining constipated into adolescence [6]. The disorder significantly impacts the quality of life, limiting routine activities and causing social and physical distress [12,13,14,15,16,17].

FC manifestations vary from mild forms, usually responsive to laxatives, fiber, and behavioral modifications, to severe cases refractory to standard medical and behavioral management and referred for surgical evaluation [18]. Of patients with FC seeking surgical assessment, 75% are struggling with fecal incontinence with only 10–30% of patients requiring a surgical intervention [18,19]. This emphasizes the importance of throughout evaluation and dedicated bowel management in these children. A structured approach to bowel management is the key to treating constipated children and is effective in 87% of adherent patients with FC [20] leading to a dramatic decrease in hospital admissions, emergency department (ED) visits, and healthcare costs [21,22].

We present a review of bowel management protocols for patients with functional constipation referred for surgical evaluation. This review includes updates on evaluation and medical and surgical management options in these children and belongs to a series of manuscripts on bowel management aspects for patients with anorectal malformations (ARMs), Hirschsprung disease (HD), spinal anomalies, and functional constipation [23].

## 2. Methods

A review of the literature published before March 2023 in the Medline/PubMed, Google Scholar, Cochrane, and EMBASE databases, including original studies, meta-analyses, randomized controlled trials, and systematic reviews, was performed focusing on manuscripts and books published over the last 5–10 years in English. Search keywords included: “bowel management”, “functional constipation”, “Botox”, “botulinum toxin”, “fecal incontinence”, “enema”, “laxatives”, “constipation”, “colorectal surgery”, and “resection”. The reference lists of the retrieved articles were checked for other relevant articles not found during the initial search. Articles providing novel insights or addressing current challenges in the field were prioritized. One hundred and eleven of the selected articles were included in the current review. The data was reported in a narrative format focusing on the recent updates in the bowel management of patients with FC and used to inform an in-depth, stepwise protocol for bowel management. The search was age limited, including patients up to 21 years of age. The section on the management of pelvic floor dyssynergia includes studies in adults to report the recent outcomes of pelvic floor physiotherapy and biofeedback that could be potentially implemented in the pediatric population.

## 3. Initial Evaluation

When consulting a patient with chronic constipation, past medical history, including prior surgical procedures, past diagnostic tests, the current stooling pattern, and bowel regimen, should be reviewed. In addition to ARMs, HD, and spinal anomalies, the potential causes of constipation include endocrine and metabolic disorders (hypothyroidism, celiac disease), medications, connective tissue disorders, milk protein intolerance, and other conditions that should be carefully addressed during the initial evaluation [24,25,26].

**Figure 1:**
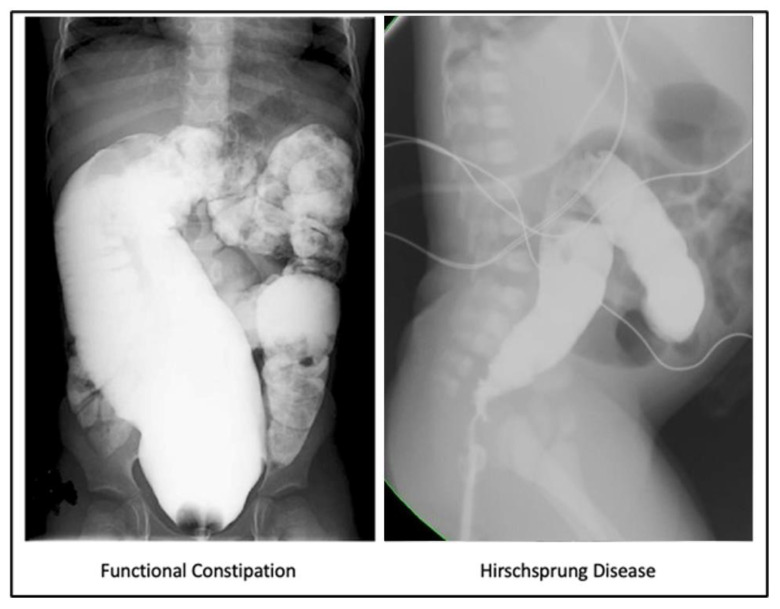
For a patient who has not had reconstructive surgery but suffers from severe constipation, a water-soluble contrast study is helpful. The characteristic image is a megarectosigmoid with dilation of the colon down to the level of the levator mechanism (on the **left**) compared to patients with Hirschsprung disease (on the **right**).

Patients with FC can present with overflow incontinence secondary to constipation that can be visualized on a contrast enema which helps assess colonic anatomy and stool passage [1]. Some gastroenterological studies do not suggest using radiology for the initial diagnosis of FC [27,28,29] with a complete medical history and throughout physical examination being sufficient for the diagnosis establishment [27,30]. However, patients with persistent constipation referred to a pediatric surgeon require other anatomic causes of constipation to be excluded with a contrast enema. Colon dilatation down to the levator muscle complex is a characteristic feature of FC on contrast enema (Figure 1), while rectosigmoid redundancy could potentially lead to poor response to medical treatments. However, if the patient responds to laxative treatment, the severity of rectosigmoid dilation was reported not to be associated with the laxative dosage required to achieve social continence [24].

Further diagnostic and treatment steps will cover the management of patients with FC referred for surgical evaluation. Examination under anesthesia (EUA) is required to assess the anorectal area for visual anomalies and anal stenosis. If the operating room time is not available, the examination can be performed in the clinic. Digital rectal examination is vital to rule out anal stenosis, dilated hemorrhoidal veins, and anal fissures that could cause chronic constipation and unpleasant defecation experience [25,26]. If a patient has a rectosigmoid index on contrast enema less than 1, Hirschsprung disease should be ruled out with anorectal manometry and/or rectal biopsy (with a full-thickness rectal biopsy remaining the gold standard) [31,32].

Since 2016, Rome IV criteria have been utilized for FC diagnosis, differentiating children with no stooling pattern (up to 4 years of age) and those who are toilet trained. According to these guidelines, patients with FC experience at least two conditions for at least one month: 1–2 defecations per week, excessive stool withholding, painful or hard defecation, large stools, and fecal impaction. When inadequately treated, incomplete colonic emptying accumulates a larger amount of stool and megacolon, leading to overflow soiling. Thus, in older, toilet-trained children, one or more soiling episodes per week and large-diameter feces obstructing the toilet are additional criteria for an FC diagnosis [11,33,34,35]. Once the organic causes of constipation are considered and cannot fully explain symptoms, and Rome IV criteria for constipation are met, the diagnosis of FC is confirmed.

## 4. Factors Affecting Continence Potential

Children with FC have normal anal sphincters, a normal spine, and no congenital anatomic diseases of the anorectal area. Fecal continence in these children depends on three main factors: (1) sphincters, (2) anal canal sensation, and (3) colonic motility, which will be addressed in the further sections of the manuscript. In addition to the anatomic characteristics and motility, other factors such as age, behavioral and neurologic concerns, socioeconomic status, and demographics can affect the patient’s likelihood of continence [18,36].

### 4.1. Age

Children with FC can face significant challenges associated with their condition [37,38]. In comparison to other colorectal anomalies, FC develops as the child grows leading to severe psychosocial distress for both the patients and their caregivers. There are three time points when children are at higher risk of developing constipation: (1) introduction of solid and high-fiber food in the diet, (2) toilet training, and (3) start of school [36]. When solid food enriched with fiber is introduced to a child, the stool becomes firmer and is harder to pass, therefore, these dietary changes can cause difficulties at defecation. Toilet training, which normally occurs in children by the age of 4 years, is a significant milestone in the child’s development and is associated with the increased anxiety of the child struggling to achieve independence and justify their parents’ expectations [39,40,41]. As school education starts, children face new challenges associated with defecation exaggerated by social pressure in the novel environment. Insecurity and bullying at school can lead to depression and low self-esteem and need to be addressed [13,42,43].

**Figure 2:**
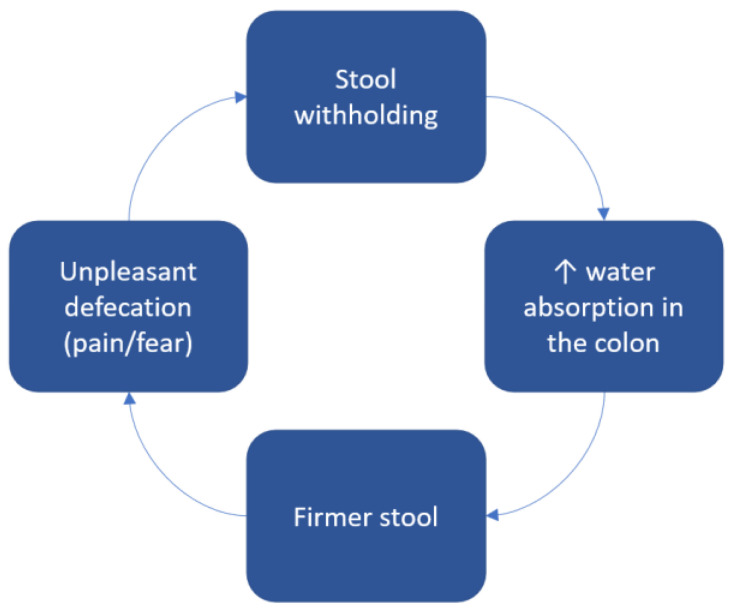
Pathogenesis of withholding behavior in patients with constipation. Children that have had frightening or painful defecations start holding the stool to avoid this unpleasant experience. The longer the stool stays in the colon, the more water is absorbed, leading to firmer stool, which is even more difficult to pass. In this way, the more a child tries to hold the stool in, the more difficulties they experience during defecation.

All the factors described above lead to high emotional stress associated with defecation. With a desire to avoid the frightening or painful experience, the child would try to decrease the frequency of defecations and start holding the stool in [44]. The stasis in the colon leads to increased water absorption, and therefore firmer feces that are more difficult to pass through. These events result in a vicious cycle where the longer the child tries to avoid defecation, the more painful the defecation becomes (Figure 2) [45]. Addressing the psychosocial concerns is crucial for the development of a trusting patient–family physician relationship, promotion of open conversation about the disorder, and adherence to the treatment plan [46,47].

### 4.2. Neurologic and Psychiatric Issues

Of patients with FC referred to a pediatric surgeon, 26–38% are neurodiverse with an associated neurologic (12%) or psychiatric (26%) diagnosis or behavioral concerns [48,49,50]. The statement that these conditions can affect the continence potential remains controversial [18,51,52] with only developmental delay having been reported to be associated with a worse prognosis for continence [18]. Other diseases, such as attention deficit hyperactivity disorder (ADHD), depression, and obsessive-compulsive disorder have not been proven to affect the outcomes [18]. Anxiety and developmental delay have been reported to be associated with increased use of antegrade continence enemas (ACEs) in FC patients [52]; however, there is no significant difference in the time required to achieve continence between neurodiverse and neurotypical patients [53]. Recently, Seidler et al. reported that participation in a dedicated BMP led to significant improvement of FC (up to 90%) and urinary continence (up to 91%) in both neurodiverse and neurotypical patients [50].

### 4.3. Socioeconomic Factors

Socioeconomic factors such as public insurance, lower education level, and lower income are associated with a higher constipation prevalence [13,54,55,56,57] and a higher risk of overflow incontinence in patients with FC [18].

The child–parent relationship is another important factor affecting the outcomes. Niu et al. emphasized the importance of family communication where the parents blaming a child for having accidents, a conflict between the parents, an authoritarian or doting parenting style, anxiety or temper control in children, and anxiety or depression in the parents predicted the development of constipation in children of preschool age [57,58]. Poor bowel habits and the child’s picky eating are also associated with an increased risk of FC [58]. Psychosocial assessment of the families could be beneficial to address the factors affecting the child’s constipation [57], discuss the possible changes in the family environment and possibly improve the outcomes.

## 5. Stepwise Bowel Management Protocol

**Figure 3:**
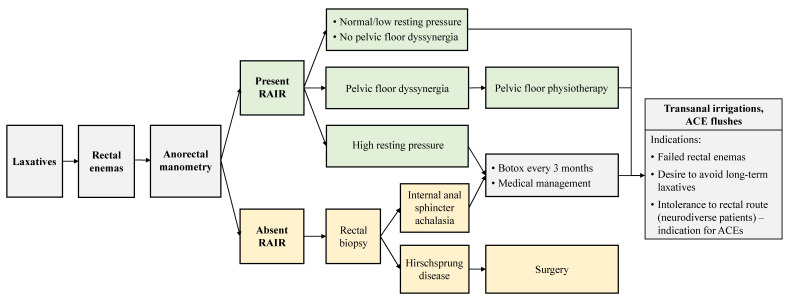
Algorithm of evaluation and treatment of patients refractory to medical therapy with laxatives and/or rectal enemas based on the anorectal manometry results. ACE—antegrade continence enema; RAIR—rectoanal inhibitory reflex.

The goal of treatment is to empty the colon daily and reduce symptoms associated with constipation such as overflow fecal incontinence. Most patients with FC can be successfully treated with behavior modification and laxative medication [11]. Even though it has been hypothesized that dietary modifications can affect constipation, there are limited data supporting the role of nutrition in the management of these children [59]. The stepwise bowel management protocol for patients with FC referred to a pediatric surgeon is demonstrated in Figure 3.

### 5.1. Laxatives

Initially, treatment can include stimulant laxatives (with or without water-soluble fiber) if the child has soft stool, while patients with hard or dense stools may benefit from osmotic laxatives. Long-term use of senna-based stimulant laxatives was proven to be effective and safe in pediatric patients [60] inducing fluid secretion into the bowel lumen as well as directly stimulating colonic contractions [61]. Bisacodyl has been reported to be effective in 57% of patients, with 55% being successfully weaned off the medication at the median follow-up of 1.5 years [62]. In 8% of patients, bisacodyl can lead to abdominal pain and fecal incontinence [62].

Initially, a 7-day trial with stimulant laxatives is performed to assess the evacuation of stool based on radiographic findings [21]. The start dose of laxatives is defined empirically, taking into consideration the degree of colonic dilation and the child’s weight. [63]. After the regimen is started, its effectiveness is assessed based on the stooling pattern and the abdominal X-rays and adjusted as needed [21]. Further information on the organization of a structured bowel management program can be found in a related manuscript “Pediatric Bowel Management Options and Organizational Aspects” [64].

Controversy exists regarding what should be considered a “failure” of medical management. The criteria are defined as (1) previous participation in a structured bowel management program, (2) persistent severe constipation, (3) failure to pass stool with abdominal distention on high laxatives doses. Patients who meet these criteria are switched to mechanical treatment options (rectal enemas, transanal irrigations, or antegrade continence enemas) [63].

### 5.2. Rectal Enemas

If a patient experiences abdominal cramping due to overstimulation of the colon in response to stimulant laxatives or is irresponsive to laxatives, the child is switched to rectal enemas. Importantly, mechanical emptying of the colon (rectal enemas, transanal irrigations, antegrade flushes) must not be used with laxatives as enemas empty the colon, while laxatives lead to further contractions of the bowel and result in leakage of stool. Some patients do not respond to rectal enemas and require further motility assessment to guide treatment.

### 5.3. Assessment of Anorectal Motility

Passage of stool through the colon and the anorectal region requires coordinated contraction of smooth muscles of the gastrointestinal tract to allow for propulsion of stool. Disruption of neuromotor regulation leads to impaired passage of stool and can be revealed on manometry studies of the anorectal region and/or colon.

**Figure 4:**
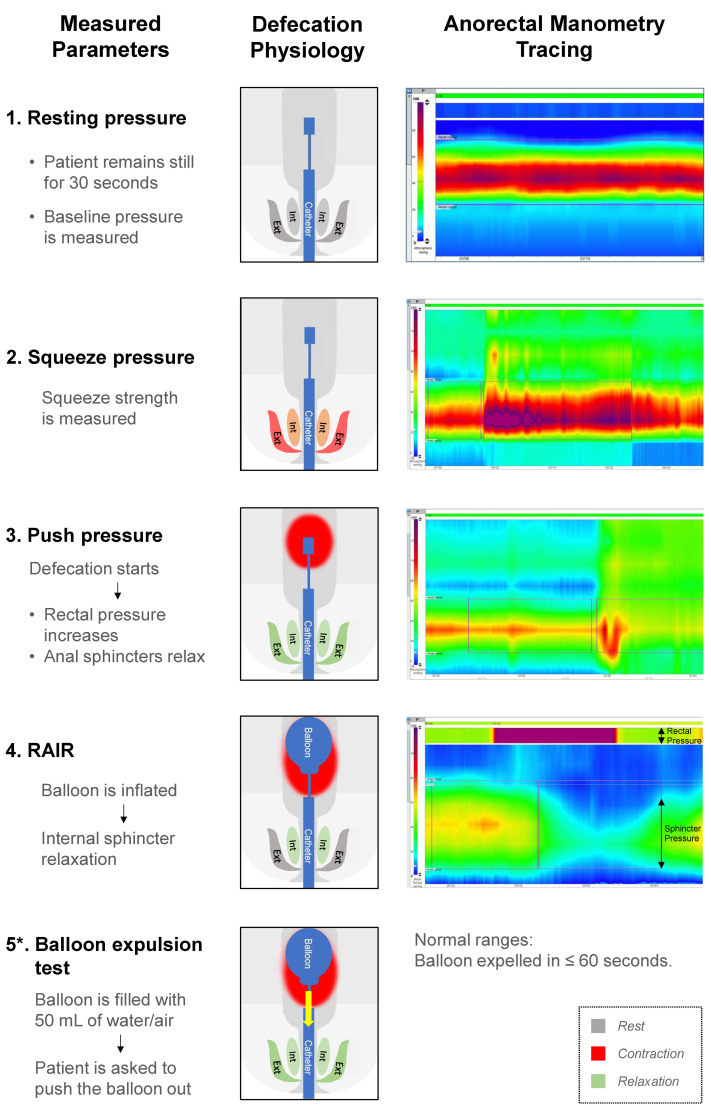
Anorectal manometry: the technique, steps of physiologic defecation tested, and examples of tracing reflecting the tested parameters. * Balloon expulsion test can be performed at the time of the anorectal manometry.

Children unresponsive to medical management with rectal enemas require further evaluation of anorectal function using anorectal manometry (AMAN) which provides information about the rectoanal inhibitory reflex (RAIR), sphincter resting pressures, dynamics of defecation, and rectal sensation (Figure 4) [42,65,66] to determine further management strategy. The procedure takes approximately 30 min, during which the patient’s cooperation in following the instructions is required.

In neurodiverse patients who cannot follow instructions, AMAN can be performed under sedation and allows assessment of only the RAIR and resting sphincter pressures [65,67,68]. The same parameters can be evaluated in children who are not toilet trained (younger than 4 years) and have not learned to coordinate defecation.

#### 5.3.1. Non-Relaxing Sphincters

**Figure 5:**
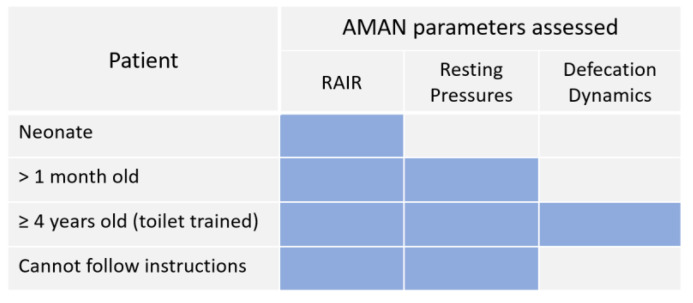
Parameters assessed on anorectal manometry based on the age and ability to follow the instructions. AMAN—anorectal manometry; RAIR—rectoanal inhibitory reflex.

AMAN is a useful screening tool for HD in children of all ages [69,70,70,71] that makes it possible to avoid a more invasive rectal biopsy that can lead to bleeding, perforation, or infection [70]. An absent RAIR indicates a full-thickness rectal biopsy to differentiate HD [31,65,69] from internal anal sphincter achalasia in which the anal sphincters fail to relax despite the presence of rectal ganglion cells [31,72,73]. Resting pressures can be measured in patients after the first month of life as the anal sphincter progressively matures in the first weeks of life [70] (Figure 5).

Patients with high resting pressures, as well as children with internal anal sphincter achalasia, require botulinum toxin injections to allow for sphincter relaxation [65,74,75,76,77,78]. Given the possible need for a rectal biopsy and botulinum toxin injections after manometry, the EUA, AMAN, rectal biopsy, and botulinum toxin injections can be performed under the same anesthesia [31].

The dosage of the toxin varies in the literature from 12 to 200 units or 6 U/kg, depending on the age of the patient and the surgeon’s preferences [77,79,80,81,82]. At our institutions, 100 units are circumferentially injected into the anal sphincter at the level of the dentate line [31,76], avoiding the anterior rectal wall to prevent damage to the urethra. Further studies are required to define the optimal dose calculation.

The effect from botulinum toxin has been reported to dissolve after 3–6 months [10] indicating repeat injections as needed; however, in some cases, it can remain for longer than one year [82]. If the symptoms do not improve, the frequency of the injections can be increased, or another brand of botulinum toxin changed. Botulinum toxin injections can lead to fecal incontinence, which resolves within a week [82].

#### 5.3.2. Pelvic Floor Dyssynergia

Once the child is toilet trained (usually by the age of four), defecation dynamics can be assessed. Pelvic floor dyssynergia is uncoordinated contractions between the pelvic floor and abdominal muscles during defecation leading to constipation and difficulty with defecation. In order to help the patients train to contract and relax the external anal sphincter, improve rectal sensation, and coordinate contractions of the internal and external anal sphincters, pelvic floor physiotherapy (PFPT) is performed [63,65,83,84]. There is limited evidence on the use of botulinum toxin injections in this group of patients [85,86,87,88]; thus, they cannot be currently recommended as a part of a standardized treatment protocol.

Until recently, there were limited data available on the outcomes of PFPT on FC management. A multicenter randomized controlled trial showed PFPT to be more effective than laxative treatment without PFPT in children with FC. Outcomes include increased cessation of laxative use as well as improvement of patient and parental quality of life [89]. Another recent double-blind randomized study showed a significant impact of pelvic floor physiotherapy combined with interferential electrical stimulation on constipation treatment when compared to the non-PFPT group (88% vs. 43%) [90].

Biofeedback is a method of PFPT using visual or other sensory guidance performed either in the office or at home [91] aiming to teach the patients with a satisfaction rate of up to 91% [84,92], improvement of clinical characteristics [93,94,95,96,97,98,99,100] and manometric parameters leading to increased rectal sensation, improved RAIR and defecation dynamics [93,94,95]. The American Neurogastroenterology and Motility Society and the European Society of Neurogastroenterology and Motility recommend biofeedback for the management of constipation in patients with pelvic floor dyssynergia and soiling [65].

### 5.4. Transanal Irrigations and Antegrade Continence Enemas

If the treatment described above is ineffective, transanal irrigations (TAIs) or antegrade continence enemas (ACEs) are initiated. Other indications for these management options include the patient and family’s desire to avoid long-term laxative treatment and inability to tolerate rectal enemas, for example, in neurodiverse patients with behavioral disorders and autism [63], which makes ACE flushes preferable [63,101].

Transanal irrigations are performed using a rectal catheter through which the solution is administered into the bowel under pressure [102]. Of all groups of colorectal patients suffering from fecal incontinence, children with FC have the best response to transanal irrigations (TAIs) [103] with their effectiveness highly dependent on the parents’ training to perform the irrigations at home that increases adherence to the regimen [104]. TAIs have been reported to be effective in patients with FC [105,106] and associated with an 86% parental satisfaction rate [107].

ACE was proven to be an effective treatment option in children with FC including those with pelvic floor dyssynergia and neurological or behavioral conditions [53,83]. At a median follow-up of 2.5 years, 90% of FC patients were reported to be socially continent with 15% of children transitioning to laxatives with no further need for ACE flushes [83]. Of patients with pelvic floor dyssynergia, ACEs are used as an addition to or as a next step after pelvic floor physiotherapy with 92% of these children achieving continence [83]. Jacobs et al. reported 1.3–1.7 months being required for patients with FC presenting with soiling to achieve continence with long-term success maintained in 90% of the children. The time required for constipation to improve also did not depend on their underlying neurological or behavioral disorders [53]. For further information on ACE procedure updates, please refer to the related article on organizational aspects of a bowel management program [64].

### 5.5. Assessment of Colonic Motility

If antegrade flushes are ineffective for achieving bowel management goals, colonic motility can be assessed using colonic manometry (CMAN) [108]. If colonic manometry is not available, nuclear scintigraphy or sitz mark study can be performed to assess colonic transit; however, these tests have several limitations and lack a standardized protocol for the assessment of colonic motility [109,110]. Sitz marker study is associated with increased radiation exposure and multiple visits required for assessment of colonic motility, while colonic scintigraphy is an expensive test with limited availability across institutions [109]. There are three possible results of the colonic motility evaluation:

(1) Normal motility with the presence of high-amplitude propagated contractions (HAPCs) throughout the colon;
(2) Segmental dysmotility (usually sigmoid);
(3) Diffuse colonic dysmotility (no HAPCs in the entire colon).

Until recently, the preferred timing of CMAN was at the time of AMAN to perform a full assessment of anorectal and colonic motility to guide further management with segmental dysmotility being the most common type (80% of patients with refractory functional constipation) and indication for sigmoid resection [101]. In 2022, Ahmad et al. reported that 92% of patients with segmental dysmotility respond to ACE flushes and might not require a segmental resection [111], which changed the evaluation protocol. With this new data, even if a patient is diagnosed with dysmotility (i.e., segmental dysmotility in most cases), a resection will be required in only 8% of patients, which makes colonic motility assessment more reasonable only in patients who fail antegrade flushes.

### 5.6. Surgical Strategy

**Figure 6:**
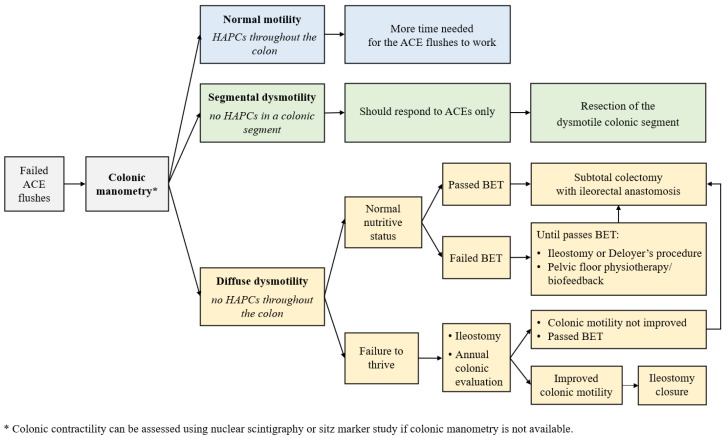
Algorithm of evaluation and treatment for patients with refractory functional constipation. Planning of surgical management in patients who failed antegrade flushes based on colonic motility. ACE—antegrade continence enema; BET—balloon expulsion test; HAPC—high-amplitude propagated contraction.

Failure of medical management with laxatives and mechanical treatment options (rectal enemas, transanal irrigations, and antegrade continence enemas) is the main indication for a surgical procedure in patients with FC [112] followed by persistent fecal incontinence and significant rectosigmoid dilation [63]. The wide range of surgical procedures performed for refractory FC has been described in the literature including a diverting ostomy, sigmoid resection with or without a simultaneous ACE procedure, Deloyers procedure, pull-through variations, proctocolectomy with an ileoanal anastomosis, colon resection with an ileorectal anastomosis [63,101,113,114]. The focus of this manuscript is the current protocol used in this patient group as demonstrated in Figure 6.

Normal colonic contractility and segmental dysmotility, as described above, are managed with ACE flushes, with sigmoid resection required in 8% of patients with segmental dysmotility.

Patients with diffuse colonic dysmotility are very challenging to manage. Recently, the strategy of their management has been broadly discussed among the leading pediatric colorectal centers. One of the key aspects of surgical interventions in FC children is the preservation of the rectum, and thus, the continence mechanism which prevents postoperative fecal incontinence [114].

There is an ongoing study on the role of the balloon expulsion test as a preoperative tool to define the optimal surgical treatment for patients with diffuse dysmotility [115]. The balloon expulsion test (BET) is a tool utilized for the diagnosis of defecation disorders such as pelvic floor dyssynergia and pelvic outlet obstruction [116,117,118,119]. The test makes it possible to assess the patient’s ability to expel a balloon that imitates the stool. While the technique and criteria for passing or failing the test vary throughout the studies [117,118,120,121,122]. The balloon is inserted into the rectum, filled with 50 mL of water or air (Figure 4), and the patient is asked to expel it as fast as possible with the goal being to do so within 60 s [115].

It is hypothesized that if the child can expel the balloon, they will be able to evacuate stool after a colonic resection [115]. For this reason, subtotal colectomy with an ileorectal anastomosis is hypothesized to be the optimal surgical procedure for these children [115]. Pelvic floor dyssynergia, which is also associated with the patient’s ability to pass the BET, can play a role in the ability to achieve success after the resection and needs further investigation.

**Figure 7:**
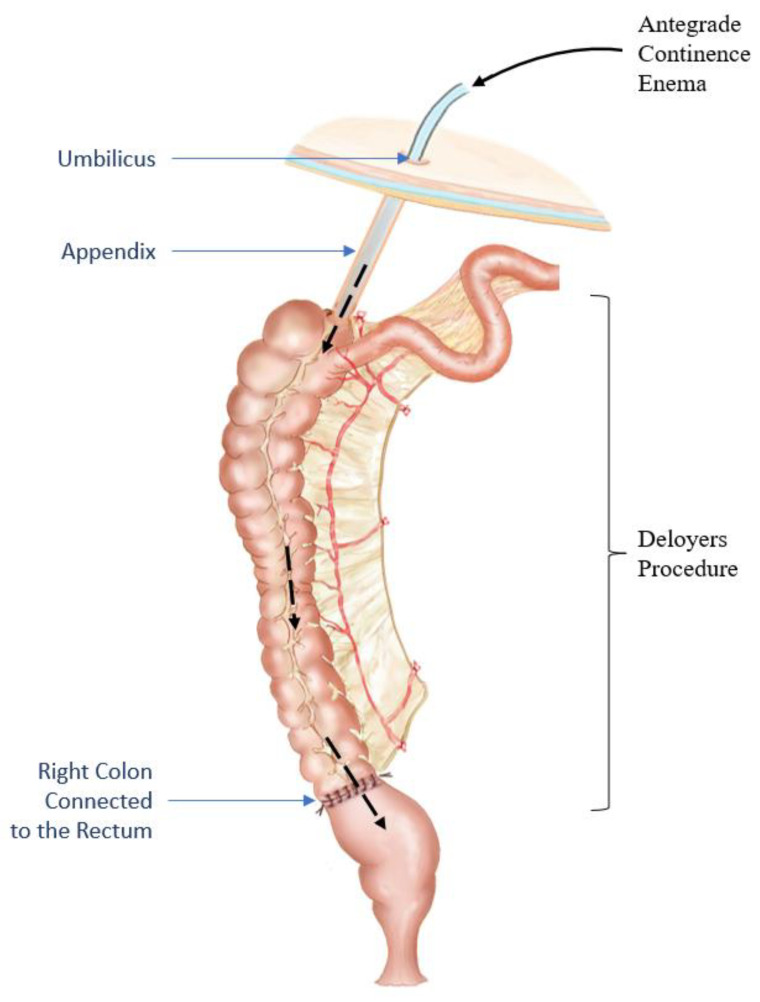
Derotation maneuver (Deloyers procedure) performed in patients who fail the balloon expulsion test with a simultaneous antegrade continence enema procedure (ACE) in the right colon. ACE flushes help to mechanically empty the colon until the patient trains to pass the stool out (i.e., pass the BET). Modified from Jouvin, I.; Pocard, M.; Najah, H. Deloyers procedure. *J Visc Surg.* **2018**, *155*, 493–501 [124].

While passed BET is an indicator of appropriate defecation dynamics, patients who fail the BET require additional help to pass the stool out. For this purpose, they undergo a Deloyers procedure (derotation maneuver) with a simultaneous ACE procedure that allows for antegrade flushes to mechanically empty the preserved right colon (Figure 7). A similar technique flushes has been described for patients with segmental dysmotility and megarectosigmoid who underwent a sigmoid resection with an ACE creation that made it possible to decrease the need for laxatives in these children [101,123]. After the Deloyers procedure, the patient is referred to pelvic floor physiotherapy or biofeedback to train coordinated defecation. Once the patient can pass the BET, a subtotal colectomy and ACE takedown can be performed [115]. If a Deloyers procedure with ACE flushes is ineffective or presents with failure to thrive (usually under 3 years of age), an ileostomy is performed [63,115] with an annual colonic motility assessment [63].

### 5.7. Other Treatment Options

Anal dilations, internal anal sphincter myectomy, and sacral and tibial nerve stimulation were described as alternative options of treatment in patients with retractable FC. Anal dilations have not been proven to be effective when compared to a placebo [68,125]. There was no difference in outcomes between myectomy and botulinum toxin injections [126], while myectomy leads to soiling and thus is not widely used [63].

Until recently, there has been poor evidence on the use of sacral and percutaneous tibial nerve stimulation (SSN, PTNS) in children with FC. Since the last review covering stepwise management of refractory FC was published [63], there have been two randomized trials on SNS in patients with constipation conducted [127,128]. The response rate was 57–60% at 6 months [128] and 55% at a 1-year follow-up [127]. However, there were complications after the SNS mentioned in the literature, including severe infections (20%), pain associated with device implementation (25%), and non-compliance (5%) [127,128] with a strong persistent placebo effect associated with SNS suggested [128]. Abreu et al. conducted a randomized clinical trial on the effectiveness of parasacral transcutaneous nerve stimulation in children with neurogenic bladder and bowel [129]. The results suggested a positive impact of the intervention when compared to a control group [129]. A randomized, double-blind, controlled that demonstrated PTNS in combination with PFPT as an effective method of constipation management in patients with dyssynergic defecation [130].

## 6. Conclusions

On referral for surgical consultation and management, patients with functional constipation refractory to medical and behavioral management should undergo evaluation with a contrast study, anorectal examination under anesthesia, anorectal manometry, and a full-thickness rectal biopsy. Anal botulinum toxin injections are administered in those with internal sphincter achalasia or high resting anosphincteric pressures. Bowel management options include laxative medications, rectal enemas, transanal irrigations, and antegrade flushes. If medical management does not achieve treatment goals, then patients require an assessment of colonic motility and possible colonic resection. Further studies are required to determine the outcomes of the existing surgical treatment options and the role of the balloon expulsion test in preoperative planning.

## Data Availability

No new data were created or analyzed in this study. Data sharing is not applicable to this article.
